# Serotonin Neuronal Function from the Bed to the Bench: Is This Really a Mirrored Way? 

**DOI:** 10.1523/ENEURO.0021-19.2019

**Published:** 2019-06-04

**Authors:** Adeline Etievant, Thorsten Lau, Guillaume Lucas, Nasser Haddjeri

**Affiliations:** 1 Integrative and Clinical Neurosciences EA481, University of Bourgogne Franche-Comté, Besançon F-25000, France; 2 Department of Translation Brain Research, Central Institute for Mental Health, Medical Faculty Mannheim, Heidelberg University; 3 HITBR Hector Institute for Translational Brain Research gGmbH, Mannheim J5 68159, Germany; 4 German Cancer Research Center (DKFZ), Heidelberg INF280, Germany; 5 Institut National de la Santé et de la Recherche Médicale, University of Bordeaux, Neurocentre Magendie, Physiopathologie de la Plasticité, Neuronale, Unité 1215, Bordeaux F-33000, France; 6 Univ Lyon, Université Claude Bernard Lyon 1, Inserm, Stem Cell and Brain Research Institute U1208, Bron F-69500, France

**Keywords:** firing activity, induced neurons, induced pluripotent stem cells, reprogramming, serotonin, translation

## Significance Statement

Induced pluripotent stem cells (iPSCs) offer a great opportunity to recapitulate both normal and pathologic development of brain tissues. Recently, three research teams have developed human-PSC technology and direct somatic cell reprogramming to allow induction of human serotonin (5-hydroxytryptamine; 5-HT) neurons *in vitro*. While preclinical studies have repeatedly shown that 5-HT suppresses 5-HT neuronal firing activity, one group has tested the effect of 5-HT on the neuronal activity of those 5-HT-like cells and found a paradoxical excitatory action of 5-HT. Here, we argue that few cautions in translational interpretations have to be taken into account. Nonetheless, using patient-derived cells for generating disease relevant cell types truly offers a new and powerful approach for investigating mechanisms playing fundamental roles in psychiatric disorders.

## 

Disease modeling by direct reprogramming into desired cell types represents a new huge challenge. Induced pluripotent stem cells (iPSCs), cells reprogrammed from human somatic cells, offer a great opportunity to recapitulate both normal and pathologic development of brain tissues and may as well provide essential strategies toward cell-based therapy of neuropsychiatric diseases (Vadodaria et al., 2018). Successfully, in 2016, three research teams have developed human-PSC technology (Lu et al., 2016) and direct somatic cell reprogramming (Vadodaria et al., 2016a; Xu et al., 2016) to allow induction of human serotonin neurons *in vitro* for the first time (for review, see Vadodaria et al., 2016b).

Remarkably, Lu et al. (2016) have demonstrated the accurate timely regulation of WNT, SHH, and FGF4 signaling pathways during the serotonergic (5-HT) neuron differentiation and generated an enriched population of 5-HT neurons from human embryonic stem cells (ESCs) and iPSCs. These human 5-HT neurons not only express specific biomarkers (TPH2, 5-HT, GATA3, GATA2, FEV, LMX1B, SERT, AADC, and VMAT2) but also show electrophysiological activities and release 5-HT in response to stimuli in a dose-dependent and time-dependent manner (Lu et al., 2016). Subsequently, this group further analyzed the features of human iPSCs-derived 5-HT neurons both *in vitro* and *in vivo*. They found that these human 5-HT neurons are sensitive to the specific neurotoxin 5,7-dihydroxytryptamine *in vitro*. After being transplanted into new-born mice, the cells not only expressed their typical molecular markers but also showed the migration and projection to the cerebellum, hindbrain, and spinal cord. Clearly, the obtained human iPSCs-derived neurons exhibit the typical features of the 5-HT neurons in the brain (Cao et al., 2017). As observed *in vivo*, a recent study also described selective serotonin reuptake inhibitor (SSRI)-dependent elevation of extracellular 5-HT concentrations, caused by the antidepressant citalopram exposure of human iPSC-derived 5-HT neurons (Vadodaria et al., 2019).

Accordingly, somatic cells were also shown to be directly converted to functional neurons (directly induced neurons) through ectopic expression of neural conversion factors. Consequently, dopaminergic, cholinergic, or striatal medium spiny neurons have been recently generated directly from human fibroblasts by using forced expression of lineage-specific transcription factors acting during brain development (Miskinyte et al., 2017). Therefore, Xu et al. (2016) demonstrated the efficient conversion of human fibroblasts to serotonin induced neurons following expression of the transcription factors Ascl1, Foxa2, Lmx1b, and FEV. The authors have examined the trans-differentiation that was enhanced by p53 knock-down and suitable culture conditions (including hypoxia, which was shown to increase the yield of 5-HT neurons). Importantly, Xu et al. (2016) verified that serotonin induced neurons were able to express markers for mature 5-HT neurons, presented Ca2+-dependent 5-HT release and selective 5-HT uptake, and exhibited spontaneous action potentials and spontaneous excitatory postsynaptic currents. Surprisingly however, bath application of 5-HT significantly increases the firing rate of spontaneous action potentials. In parallel, Vadodaria et al. (2016a) showed that overexpressing a different combination of 5-HT phenotype-specific transcription factors (NKX2.2, FEV, GATA2, and LMX1B) in combination with the neuronal transcription factors ASCL1 and NGN2 directly and efficiently generated 5-HT neurons from human fibroblasts. Induced 5-HT neurons showed increased expression of specific serotonergic genes known to be expressed in raphe nuclei and displayed spontaneous action potentials, released serotonin *in vitro* and functionally responded to SSRIs.

Noticeably, the results from Xu and co-workers on the functional effect of 5-HT on spontaneous action potentials of induced 5-HT neurons appear to be in discrepancy with all the preclinical data obtained so far. Indeed, animal studies, mostly conducted in rodents, have demonstrated that this neurotransmitter exerts an inhibitory influence on the firing activity of mature 5-HT neurons (for review, see Blier and El Mansari, 2013). 5-HT neurons exist in nearly all animal taxa, from the invertebrate nervous system to mammalian brains. The 5-HT system in the vertebrate brain is implicated in various behaviors and diseases. In mammals, the cell bodies of 5-HT neurons are located in the brainstem, near or on the midline. The dorsal raphe nucleus (DRN) contains ∼50% of the total 5-HT neurons in both rat and human CNS (Piñeyro and Blier 1999). In rodents, the 5-HT-containing cells have been shown to exhibit a slow (1–2 Hz) and regular firing rate, with a long-duration positive action potential. This regular discharge pattern results from a pacemaker cycle attributed to a Ca^2+^-dependent K^+^ outward current. The depolarization is followed by a long afterhyperpolarization (AHP) period, which diminishes slowly during the interspike interval. During the depolarization, extracellular Ca^2+^ enters the neuron via a voltage-dependent Ca^2+^ channel activating a K^+^ outward conductance leading to an AHP. Ca^2+^ is then sequestered/extruded and the AHP diminishes slowly. When the membrane potential reaches the low-threshold Ca^2+^ conductance, a new action potential is triggered (Piñeyro and Blier 1999). Around five decades ago, Aghajanian et al. (1970) were the first to assess, electrophysiologically in anesthetized rodents the effects of monoamine oxidase inhibitors (MAOIs), the first class of antidepressant medications, on the firing activity of single, serotonin-containing neurons of the midbrain raphe nuclei. All MAOI tested caused depression of raphe unit firing rate by increasing endogenous 5-HT and such suppressant effects were prevented by prior treatment with an inhibitor of 5-HT synthesis. Similarly, *in vitro* and *in vivo*, direct application of exogenous 5-HT suppresses 5-HT neuronal firing activity (Piñeyro and Blier 1999). Numerous rodent studies have shown that this net effect of 5-HT is mediated via the activation of somatodendritic 5-HT_1A_ autoreceptors (for review, see Piñeyro and Blier 1999). This 5-HT_1A_ autoreceptor receives an increased activation by endogenous 5-HT at the beginning of a treatment with a SSRI or a MAOI and, consequently, a decreased 5-HT neuronal firing activity is obtained. Indeed, when activated by 5-HT, G_αi/o_-coupled 5-HT_1A_ autoreceptors trigger a strong reduction of 5-HT impulse flow through the opening of inwardly rectifying K^+^ channels and the inhibition of voltage-dependent Ca^2+^ channels (Piñeyro and Blier 1999). By reducing pacemaker firing, 5-HT_1A_ autoreceptors regulate 5-HT levels both locally in the DRN and in terminal projection regions (Courtney and Ford, 2016). As the SSRI or MAOI treatment is prolonged, the 5-HT_1A_ autoreceptor desensitizes and firing activity is restored in the presence of the SSRI or MAOI. This adaptive change has been proposed to underlie, at least in part, the delayed therapeutic effect of SSRI or MAOI in major depression (Piñeyro and Blier 1999). However, only very few studies have been conducted in humans to directly address the role of 5-HT_1A_ autoreceptors on 5-HT neuronal activity. One of the reasons resides in the small size of the DRN, which renders it virtually invisible for MRI-based *in vivo* imaging studies (Sibon et al., 2008). Interestingly still, human EEG studies have reported that the stimulation of presynaptic 5-HT_1A_ receptors induces a shift of the frequency spectrum (McAllister-Williams and Massey, 2003), an effect reflecting the inhibitory action of these receptors on 5-HT activity (Seifritz et al., 1996, 1998). More recently, clinical studies have shown that the 5-HT_1A_ agonist buspirone produces a more pronounced shift in medication-free depressed patients, confirming the hypothesis that at least some depressive disorders may be related to an abnormally enhanced functional status of 5-HT_1A_ autoreceptors, leading to a hypo-function of the 5-HT system (McAllister-Williams et al., 2014). Also of note, several PET studies have shown that an enhanced binding potential at DRN 5-HT_1A_ sites correlates with a reduced 5-HT transmission within the amygdala, thus providing indirect, but strong evidence, that these receptors inhibit terminal 5-HT release (Fisher et al., 2006). Clearly, the reason of the discrepant electrophysiological findings mentioned above appears to be puzzling. For that reason, the net effect of 5-HT on spontaneous action potentials of induced 5-HT neurons, obtained from both Lu et al. (2016) and Vadodaria et al. (2016a), should be extremely interesting to be assessed and compared. Indeed, a role of the chosen transcription factors for this opposing electrophysiological result cannot be fully ruled out (Vadodaria et al., 2018). The different combinations of transcription factors employed may cause differential maturation stages of induced 5-HT neurons. In rodent, the 5-HT_1A_ autoreceptor-mediated inhibition was shown to vary with age and was absent/reduced until Postnatal 21 (Rood et al., 2014). Xu and co-workers employed the transcription factor Ascl1, involved in rostral and caudal neurogenesis of 5-HT neurons, Foxa2, activated by sonic hedgehog signaling to induce 5-HT neuronal fate by suppression of ventral motor neuron generation, as well as Fev and Lmx1b, which are essential for the expression of the 5-HT neurochemical phenotype (Kiyasova and Gaspar, 2011). In contrast to this, Vadodaria and co-workers established generation of induced 5-HT neurons by overexpression of the 5-HT phenotype-specific transcription factors Fev, Lmx1b, Gata2, and Nkx2.2. The latter being discussed as having a cluster-specific function in 5-HT neurogenesis (Kiyasova and Gaspar, 2011). Therefore, an excitatory action of 5-HT may reflect differential maturation stages of induced 5-HT neurons, and *in vitro* maturation may be enhanced by forced expression of a larger number of neuronal and 5-HT specific transcription factors. Actually, a thorough examination of the supplementary data provided by Xu et al. (2016) indicates that even when considered mature (i.e., >46 d old), their induced 5-HT neurons display a resting membrane potential remaining as high as –42 mV, a value quite remote from those classically measured *in vivo* in preclinical studies, i.e., below –60 mv (Liu et al., 2002). Another possibility would reside in the fact that the protocol chosen by Xu and co-workers triggered a modified maturation of 5-HT_1A_ autoreceptors, leading to an alternative coupling of these receptors and preventing them to activate the G_αi/o_ subunit. In this context, the use of Patch-Seq (Fuzik et al., 2016), a recent method for obtaining full transcriptome data from single cells after whole-cell patch-clamp recordings of induced 5-HT neurons, should be very helpful to provide critical clues of these paradoxical electrophysiological results. Finally, it has to be kept in mind that *in vivo*, 5-HT neurons are part of a mature circuitry that obviously cannot be fully recapitulated *in vitro*, which might also impair the efficacy of 5-HT_1A_ autoinhibition.

Alternatively, the discrepancy between the results of Xu et al. (2016), and those observed in rodents may be related to a differential sensitivity toward distinct kinds of 5-HT autoregulation. Indeed, it has recently been proposed that 5-HT_2B_ receptors may constitute a new class of autoreceptors that would actually be excitatory, therefore counteracting the influence of the 5-HT_1A_ ones (Belmer et al., 2018). In mice, this positive autoregulation appears to be negligible with respect to the 5-HT_1A_-related autoinhibition, requiring the use of specific 5-HT_2B_ agonists to be unmasked (Belmer et al., 2018). It remains possible that the induced 5-HT neurons obtained by Xu et al. (2016), express a higher proportion of 5-HT_2B_ receptors, rendering the net influence of 5-HT positive on them. Thus, it would be very informative to assess the excitatory action exerted by 5-HT on the spontaneous action potentials of these cells with both selective 5-HT_1A_ and 5-HT_2B_ receptor antagonists. If this latter hypothesis were to be confirmed, the next step would be to determine whether such a higher expression of 5-HT_2B_ receptors constitutes a distinct feature of human 5-HT neurons, or whether it results from the technique of induction.

In summary, even if several advantages and inconvenients can be addressed in the use of iPSCs versus induced neurons, in terms of cell source, time and cost efficiency as well as expendability (Mertens et al., 2018), all three groups have provided, the same year, important and robust data on the conversion of human cells to induced 5-HT neurons (Lu et al., 2016; Vadodaria et al., 2016a; Xu et al., 2016). In opposition to the electrophysiological results of Xu et al. (2016), preclinical studies have repeatedly shown that 5-HT suppresses 5-HT neuronal firing activity. Significantly, this inhibitory action of 5-HT is frequently related to the well described therapeutic delay of antidepressant action, has been recurrently considered as a “brake” of the antidepressant response and has initiated numerous studies on the development of new and effective therapeutic strategies (Artigas et al., 2017). Furthermore, learning more about the electrophysiological properties of human iPSC-derived 5-HT neurons will not only help to understand serotonergic autoregulation, but also significantly contribute to understanding 5-HT neuromodulation of neuronal circuits. Even if few cautions in translational interpretations have to be taken into account, as for data obtained in animal studies, using patient-derived cells for generating disease relevant cell types truly offers a new and powerful approach for investigating the genetic and cellular mechanisms that may play fundamental roles in psychiatric disorders (Vadodaria et al., 2018).

## Synthesis

Reviewing Editor: Kirill Martemyanov, The Scripps Research Institute Decisions are customarily a result of the Reviewing Editor and the peer reviewers coming together and discussing their recommendations until a consensus is reached. When revisions are invited, a fact-based synthesis statement explaining their decision and outlining what is needed to prepare a revision will be listed below. The following reviewer(s) agreed to reveal their identity: Jian Feng.

Synthesis

Both reviewers agree that the commentary provides useful discussion on the availability of human serotonergic neurons generated from pluripotent stem cells or transdifferentiation. There was a consensus that the commentary would benefit from further editing to balance the review shifting accents (less emphasis on significance of modulation of firing by serotonin) and discussing additional areas (human raphe and contrasting it with rodent). Specific guidance is provided in the appended comments from the reviewers below.

Reviewer #1

The commentary discusses the utility of human serotonergic neurons derived from pluripotent stem cells (PSCs) or by direct conversion of fibroblasts. This novel preparation offers a useful tool to complement studies in animal models. There is no perfect model system. While animal models enable invasive studies on neurons in a network, both the neurons and the network can be very different from those in the human brain. PSC-derived or directly converted human serotonergic neurons capture the same genetic composition of the donor, although the epigenome is quite different from the in vivo counterpart due to a variety of factors, including the epigenetic reprogramming process and perhaps most importantly, the lack of a network and other supporting cells (e.g. blood vessels, glia, etc.) in the brain. A more meaningful comparison would be between rodent neuronal cultures and human neurons derived from PSC or by transdifferentiation. The availability of human neurons will make studies on rodent neuronal cultures much less informative. However, it is unclear at present how information gathered from human neurons and animal models can inform each other, because of the drastic differences. This reviewer holds the view that both approaches have their unique advantages and disadvantages. Scientific research has been very much like blind people trying to figure out what the elephant looks like. It is only when many different approaches are combined, can we generate a more realistic rendition of the elephant.

With this perspective, I find the commentary unnecessarily dwelling on one single discrepancy regarding the effect of serotonin on the firing frequency of serotonergic neurons. In induced serotonergic neurons from Xu et al., serotonin increases firing. In many previous studies in rodents, serotonin reduces firing of serotonergic neurons, presumably through 5HT1A autoreceptors. However, a recent work shows that activation of 5HT2B receptors directly increases the firing frequency of Pet1-positive serotonergic neurons (Belmer et al. Neuropsychopharmacology 2018, PMID 29453444). In light of this finding, there may not be a discrepancy.

Many studies can be done on these human serotonergic neurons. Xu et al. provides the initial tests on the physiological functions of such neurons. The lack of electrophysiological studies in Lu et al. and Vadodaria et al. makes the discussion on electrophysiology of serotonergic neurons highly asymmetrical, as there are lots of literatures on the electrophysiology of rodent serotonergic neurons. It would be more informative to discuss other aspects of human serotonergic neurons, where more information is available. A comparison on electrophysiology is premature at this point.

As eNeuro strives for impartiality through the use of double-blind reviews, the authors should refer to each paper in a consistent manner. Statement such as “Vadodaria et al. (2016) from Fred Gage's lab showed elegantly ...” seems rather subjective and gratuitous, when all other papers are cited without mentioning the senior authors' names or value judgment.

Reviewer #2

The authors review three recent studies describing methods for generating serotonergic neurons from human pluripotent stem cells and fibroblasts.

The authors focus on discussing the electrophysiological properties of serotonergic neurons and the excitatory effect of 5-HT on serotonergic neurons, as opposed to the autoinhibition that would be expected based on preclinical evidence.

The review is concise, straightforward and balanced, covering key aspects of the different methods, and poses a question about the (lack of) 5-HT1a induced feedback-inhibition in human in vitro serotonergic neurons, that would be expected in mature serotonergic neurons in vivo.

A main concern that needs to be discussed in more depth is the fact that most known electrophysiological properties of serotonergic neurons comes from rodent data and it would benefit the review to discuss data on human raphe as well as how rodent data relates to the properties of human serotonergic neurons.

Suggested are minor changes:

1. It is worth noting somewhere in the manuscript, or in the multiple points of reference to “known properties of serotonergic neurons” - that a majority of this information is entirely from mouse / rat studies. It would be relevant to specify that which of the described electrophysiological properties referred to are from human / rodent studies. Not just as a qualifier but to put it in context of any known differences between human neurons and mouse/rat neurons. (example - reference to the review by Pinero and Blier 1999 - mostly from rodent work?)

2. It would also help to discuss human imaging studies that would suggest 5HT mediated autoinhibition in human raphe. This would be relevant and applicable given that the papers discussed are about human serotonergic neurons in vitro. Since a main point in the review is the lack of 5HT1a mediated inhibition in human serotonergic neurons in vitro - the review would benefit from a deeper description of 5-HT induced autoinhibtion via 5-HT1A receptors - and what is known about the differences in human vs. mouse models (as a possible explanation for the discrepancy).

3. The authors aptly discuss that a “young” stage of in vitro human serotonergic neurons may explain the lack of 5ht induced inhibition. In the same context it would make sense to mention that this kind of autoinhibition may also be a property of not just mature cells, but also a mature circuit that is not fully recapitulated in vitro.

4. Vadodaria et al., is misspelled in the first paragraph. Another relevant reference is Vadodaria et al., Bioessays (review compares the three methods for generating human serotonergic neurons in detail).
